# Barriers and facilitators to a task-shifted stroke prevention program for children with sickle cell anemia in a community hospital: a qualitative study

**DOI:** 10.1186/s43058-023-00534-z

**Published:** 2024-01-15

**Authors:** Halima Bello-Manga, Lawal Haliru, Kudirat Ahmed, Samuel Ige, Hayatu Musa, Zainab Kwaru Muhammad-Idris, Binshak Monday, Abdulrashid M. Sani, Kemberlee Bonnet, David G. Schlundt, Taniya Varughese, Abdulkadir M. Tabari, Michael R. DeBaun, Ana A. Baumann, Allison A. King

**Affiliations:** 1 Department of Haematology and Blood Transfusion, Barau Dikko Teaching Hospital/Kaduna State University, Kaduna, Nigeria; 2 Department of Paediatrics, Barau Dikko Teaching Hospital/Kaduna State University, Kaduna, Nigeria; 3 Yusuf Dantsoho Memorial Hospital, Kaduna State Ministry of Health, Kaduna, Nigeria; 4 Department of Library and Information Science, Ahmadu Bello University, Zaria, Nigeria; 5 Department of Community Medicine, Kaduna State University, Kaduna, Nigeria; 6 Research Office, Barau Dikko Teaching Hospital, Kaduna, Nigeria; 7 Department of Psychology, Vanderbilt University, Nashville, TN USA; 8 Department of Pediatrics, Washington University School of Medicine, St. Louis, MO USA; 9 Department of Radiology, Barau Dikko Teaching Hospital/Kaduna State University, Kaduna, Nigeria; 10 Department of Pediatrics, Division of Pediatric Neurology, Vanderbilt University of School of Medicine, Nashville, TN USA; 11 Division of Public Health Sciences, Department of Surgery, Washington University School of Medicine, St. Louis, MO USA

**Keywords:** Sickle cell anemia, Stroke, Transcranial Doppler, Task shifting, Implementation

## Abstract

**Background:**

Children with sickle cell anemia (SCA) are at high risk for stroke. Protocols for stroke prevention including blood transfusions, screening for abnormal non-imaging transcranial Doppler (TCD) measurements, and hydroxyurea therapy are difficult to implement in low-resource environments like Nigeria. This study aimed to examine the contextual factors around TCD screening in a community hospital in Nigeria using qualitative interviews and focus groups.

**Methods:**

We conducted a descriptive qualitative study in a community hospital in Kaduna, Nigeria, using focus groups and interviews. Interview guides and analysis were informed by the Consolidated Framework for Implementation Research (CFIR) framework and the Theory of Planned Behavior. Transcripts were coded and analyzed using an iterative deductive (CFIR)/Inductive (transcribed quotes) qualitative methodology.

**Results:**

We conducted two focus groups and five interviews with health care workers (nurses and doctors) and hospital administrators, respectively. Themes identified key elements of the inner setting (clinic characteristics, resource availability, implementation climate, and tension for change), characteristics of individuals (normative, control, and behavioral beliefs), and the implementation process (engage, implement, and adopt), as well as factors that were influenced by external context, caregiver needs, team function, and intervention characteristics. Task shifting, which is already being used, was viewed by providers and administrators as a necessary strategy to implement TCD screening in a clinic environment that is overstressed and under-resourced, a community stressed by poverty, and a nation with an underperforming health system.

**Conclusion:**

Task shifting provides a viable option to improve health care by making more efficient use of already available human resources while rapidly expanding the human resource pool and building capacity for TCD screening of children with SCD that is more sustainable.

**Trial registration:**

NCT05434000.

**Supplementary Information:**

The online version contains supplementary material available at 10.1186/s43058-023-00534-z.

Contributions to the literature

- Understanding contextual factors that will affect establishment of a stroke prevention program for children with sickle cell disease (SCD) in a community hospital as opposed to an academic hospital is important to reduce morbidity and mortality in low-income countries with high burden of SCD.
- The Consolidated Framework for Implementation Research (CIFR) was used to examine how task-shifting of transcranial doppler ultrasonography (TCD) to nurses in the SCD stroke prevention program in a community hospital will be implemented.
- Task shifting can bridge the gap of human capacity shortage in resource-constrained settings by expanding the pool of personnel with skills for TCD.

## Background

Stroke is a frequent complication of sickle cell anemia (SCA) that is associated with increased morbidity and mortality [1]. Approximately 11% of unscreened and untreated children with SCA will have a stroke by 17 years of age [2]. A stroke prevention program includes screening for abnormal non-imaging transcranial Doppler (TCD), regular blood transfusion for at least 1 year, then treatment with hydroxyurea, if the TCD measure is elevated [3]. In high-resource settings, such evidence-based practices decreased stroke incidence rates by 92% among children with SCA [3–5].

Due to safety and availability, regular blood transfusion is not a viable option for primary stroke prevention in most resource-constrained settings, including Nigeria, where over 50% of the global 300,000 children born annually with SCA reside [6, 7]. Our team’s randomized controlled trial (SPRING trial) [8] demonstrated efficacy in primary stroke prevention among children with SCA. Now guidelines recommend hydroxyurea for children with SCA and abnormal TCD who live in resource-constrained settings [9].

Before the stroke prevention trials in Nigeria, no TCD screening was performed in Kaduna State, Nigeria, where ~ 20,040 children with SCA reside. After completing enrollment for the SPRING trial in 2019, we initiated TCD screening for children with SCA in our center, but only 10.2% (2057) of the eligible children were screened over 3 years. To increase the reach of TCD screening, we replicated our academic center-based stroke prevention program in a community hospital in a densely populated part of Kaduna, where most patients with sickle cell disease (SCD) seek care [10]. To address paucity in personnel that could perform TCD examination and expand the reach of TCD screening for children, nurses were trained to fill this gap. Task shifting can be defined as a shift in roles, where providers with different roles are trained to perform a single health-related task [11]. The task shifting from radiologists to nurses was successful, as nurses in the academic setting assumed the role of TCD assessments [10]. Our next step is to scale up SCD prevention programs in community hospitals in Nigeria with high burden of SCA and stroke.

To better understand how to replicate the task shifting of TCD screening and implement stroke prevention programs in community hospitals, we conducted a cross-sectional qualitative study with the health providers and leaders of the community setting from this pilot.

## Methods

### Study design, site, and study population

This descriptive qualitative study received institutional review board approval. The study was conducted at a 100-bed-capacity community hospital in Kaduna, Nigeria.

We conducted focus groups with nurses and doctors in the pediatric unit of the hospital. Nurses were eligible if they had worked in the pediatric unit for more than 3 years. Doctors were eligible if they (1) had experience managing children with SCD and (2) had worked in the community hospital for more than 1 year. We conducted key informant interviews with members of the hospital’s administrative staff who were involved in significant decisions affecting staff rotations, redeployment, and other hospital policies, including the medical director, hospital secretary, head of pediatrics, head of radiology, chief matron, deputy matron, and the hospital accountant. Inclusion was based on their positions and willingness to participate in the study. Those in managerial positions were separated from focus groups to reduce hesitancy of staff from speaking freely and to accommodate their limited availability. Focus groups and interviews were led by a PhD-level researcher with 15 years of experience. Because of confidentiality and resources, one note taker attended the focus group.

### Interview and focus group guides

The study team developed the interview and focus group guides (included as Supplemental material) using the CFIR as a guide [12]. The CFIR is an implementation science determinant framework that can be used to help understand the contextual factors of TCD screening through a menu of constructs of five domains: (1) intervention characteristics, (2) inner setting (3) outer setting, (4) characteristics of individuals, and (5) implementation process. We selected CIFR because of its multi-level determinants that guides possible implementation strategies that can exert effect on different level of implementation contexts [13, 14]. Additionally, CIFR has been successfully used as a determinant framework in understanding stakeholder perspective to task-shifting of hypertension services to nurses in Ghana and Nigeria [15, 16].

### Procedures

The study coordinator contacted eligible health care providers and hospital administrative staff to schedule focus groups and interviews based on participant availability. Each meeting began with introductions, an explanation of the study’s purpose, and a reminder that the session would be recorded. To set the stage for the group discussion and interviews, a short video on stroke in SCD was shown. Sessions lasted about 60 min, and participants were served lunch. Interviews with lasted about 45 min. Participants completed a short demographic survey detailing their work experience.

### Audio recording and transcription

Focus groups and administrator interviews were conducted in English and audio recorded using a digital recording device. Audio files were shared with the Vanderbilt University Qualitative Research Core (VU-QRC) for data analysis using secure transfer technology. The audio files were professionally transcribed  (https://www.rev.com/).

### Development of a coding system

The VU-QRC (D.G.S and K.B) managed qualitative data and analysis, led by a PhD-level psychologist with 30 years of experience. Data coding and analysis were conducted by following the COREQ guidelines [17]. A hierarchical coding system was developed and refined using the CFIR framework, the focus group/interview guides, and a preliminary review of transcripts. The top-level categories of the coding system, with major CFIR constructs, were identified and further divided from one to 12 subcategories with some subcategories having additional levels of hierarchical division. Coding category definitions and rules were written. The coding system underwent seven iterations before the final version was accepted.

### Coding the transcripts

Experienced qualitative coders established reliability using the coding system on two transcripts and resolving discrepancies. They then independently coded the remaining transcripts. Each statement was treated as a separate quote and could be assigned up to 14 different codes. Transcripts, quotations, and codes were managed using Microsoft Excel 2016 and SPSS version 27.0. Analysis was conducted using an analytic spreadsheet with all of the applied codes, the associated quotes, and any contextual text (e.g., moderator’s question) needed to understand the quote.

### Processing coded transcripts

We used an iterative inductive/deductive approach to qualitative analysis [18–21], resulting in a conceptual framework. Inductively, we sorted the coded quotes by coding category to identify higher-order themes and relationships between themes. Deductively, we were guided by the study questions and CFIR, [12, 22, 23]. Because some of the identified themes are at the provider or administrative behavioral level, we added the theory of planned behavior [24, 25] to provide more detailed understanding of the motivations, beliefs, and behaviors of the individuals involved in program implementation. The process was iterative as the conceptual framework was theoretically informed, while the specific framework content was derived from the coded qualitative data. Final iterations were reviewed by an implementation scientist and the team until all research team members agreed.

### Reflexivity summary

Authors from Nigeria completed graduate education in Nigeria and Europe. Authors from the USA include Asian Americans, a Latina, and African American with graduate educations. While most of the US team have not practiced in areas of extreme poverty, several team members have mentored or collaborated with researchers and practitioners in low research settings. The team has expertise in social, clinical and health psychology, SCD care, implementation science and healthcare. While all team members have the privilege of higher education, those who practice in Nigeria were raised in the country and have experience with delivering care with limited resources.

## Results

### Participants

The study was introduced to a total of 30 doctors and nurses in the pediatric and emergency departments, thereafter, interested individuals were invited to participate. Two focus groups were conducted at the community hospital with seven participants in one and 12 in the other (*n* = 19). The mean age of health care providers was 39 (SD 10.0). Three providers were male, and 16 were female. The average work experience was 12.8 years (SD 9.9). The first focus group had six doctors and one nurse (labeled “doctors focus group”). The second group was all nurses and nurse midwives (labeled “nurses focus group”). Four tribes were represented: Igala, Yoruba, Hausa, and Edo.

Five administrators were interviewed (*n* = 5), with a mean age of 52.8 (SD 3.3). Two administrators were male, and three were female. The average years of work experience was 21.4 (SD 7.8). Administrators included two nurses, two doctors, and one accountant. Three tribes were represented: Hausa, Yoruba, and Bajju.

### Conceptual framework

**Fig. 1:**
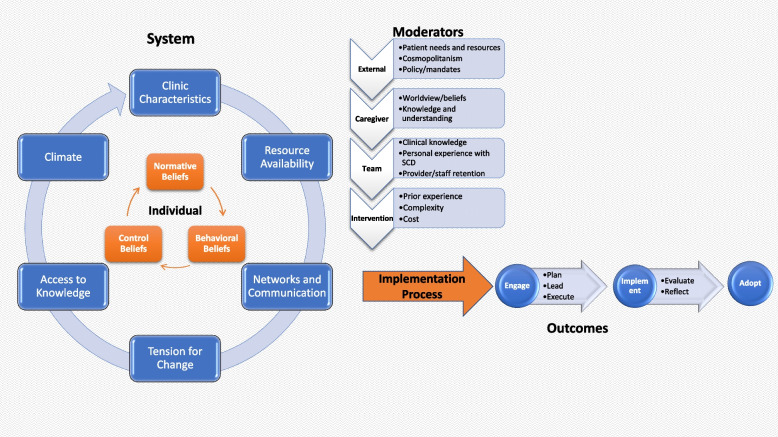
Factors influencing the adoption of TCD task shifting

Figure 1 presents the conceptual framework created following our qualitative analysis. The outer circle, largely inspired by the CFIR framework inner setting constructs [12], represents elements of the health care setting and sources of influence that drive adoption of a stroke prevention strategy implemented using TCD screening and task shifting. The inner circle, influenced by the outer circle and inspired by the theory of planned behavior [24], represents individual attitudes, beliefs, and behaviors of the health care providers involved in planning and implementing the stroke prevention program. Nested circles and arrows represent the dynamic interaction of systems and individual-level characteristics. The central area of the diagram represents modifying factors that can influence the implementation process including external factors, caregiver characteristics, implementation team characteristics, and the task shifting intervention. Joint operation of providers within a health system leads to the implementation process. All activities are related to implementing the plan, depicted as having three stages: (1) engaging, (2) implementing, and (3) adopting the prevention program.

In the following sections, each element of the framework is described and supported using quotes from coded transcripts, which are organized in Table 1. Each quote is labeled by the source of the quote.

**Table 1:** Elements of the conceptual framework supported with quotes and how they relate to CIFR domains

Elements of Conceptual Framework	Theme	CIFR Domain	Narrative	Quotes
**SYSTEM**				
	i. Clinic characteristics	Inner setting	Clinic organization is varied among the different disciplines.	-*“Like every year, they used to rotate [crosstalk].... I work in pediatric ward, of course now, and I have the knowledge, maybe by the time they do the reshufflement and I have been taken away from that ward, another set of people will have to be given that knowledge. But that will not stop if there is need for me when the need comes, I should go and help in that section.” (Nurses Focus Group)*
	ii. Resource availability	Inner setting	In some clinics staff are rotated, while in some, they are left on permanent basis.	-*“Like, we have the TB unit, the TB unit in this center is head by a nurse. The eye center, and they are permanent. Malaria unit, they are permanent nurses there. They don't shift.” (Doctors Focus Group)*
			Low remuneration, increased staff turnover, insufficient number of staff.	-*“The payment is very poor, so that is the ... like last year, we had more than 10 nurses that left this hospital, not to talk of the doctors.” (Nurses Focus Group)*
				-*“One doctor see over 400 and something patients.” (Doctors Focus Group)*
	iii. Tension for change	Inner setting	Greatest tension was around staff shortage.	“*There are times when we used to have 37 doctors here. There was a time. But they all left....The pay is better than here. They leave.” (Doctors Focus Group)*
	iv. Implementation climate	Inner setting	The participants showed evidence of the organization climate to support change and importance of creating new programs that meet the long- term needs of patients.	-*“Patients are permanent. So, you should try a solution that is lasting to them....And we know that once we start this program, we're going to identify a lot of acute patients, and more people are going to be coming.” (Doctors Focus Group)*
			Emphasis was also made on planning and to anticipate barriers and overcoming them.	-*“See what is available, how can we enhance what we have to ensure the success of the program, and the anticipated barriers that will have affect the success, so that you try and address them. So, whatever solution, you need to put it in that context....So that you are able to solve a perceived problem or an anticipated problem.” (Doctors Focus Group)*
**INDIVIDUAL**				
	i. Normative beliefs	Characteristics of the individual	Agree task shifting is a good idea.	-*“In places of this task shifting, I believe it's because of the shortage of hand among the doctors. So that's why we are shifting it to the nurses. I think if that is the case, I quite tend to agree that I think the nurses can be trained and they can do it. And if they are trained, they can take results on that. I don't see an issue with that.” (Doctors Focus Group*)
			TCD is a good intervention.	-*“Yeah, it's good to bring interventions, it should be sustained and be sustainable, and people should learn. And they should learn properly, and they should [inaudible] without any bias. As many people as they could should know what is done correctly and rightly.” (Administrator)*
	ii. Behavioural beliefs	Characteristics of the individual	How best to conduct TCD screening is to explain the importance and the procedure to parents.	-*“I know basically what you explain to the parent is that this scanning entails putting a probe on the head of your child to tell us if the pressure going to the brain is very high or not because the sickle cell we have now, it narrowed the blood [vessel] going to the brain. So, as it narrowed, the pressure going into the brain of your child is very, very high. And if we leave it like that, it will expose your child to having stroke.” (Doctors Focus Group)*
			Train nurses and supervise them.	-*“No, I think you can supervise maybe twice or three times. And if you're sure of their judgment and of the specific person doing it, from that time, you can trust anything. You know, this one I've seen him doing, I've seen her or seen him doing this for many days and I know that there is no problem.” (Doctors Focus Group)*
			Trust nurses on the ability to do TCD.	
	iii. Control beliefs	Characteristics of the individual	Willingness of both doctors and nurses to work together	-*“No. It's not like that. We trust them nurses working [inaudible]. And they do everything, all what doctors do, they can also do it. And if they can take care of a baby and unit, we can trust their judgment.” (Doctors Focus Group)*
				*“It will work if we put hand together” (Administrator)*
**PROCESS**				
	i. Engage	Process	Levels of engagement	
			i. At the hospital by engaging pediatricians and involving other departments.	-*“The need, especially the pediatricians, I think they should be the frontline.” (Doctors Focus Group)*
			ii. At the ministry by engaging the policymakers.	-*“This is a policy now; policies are done from the ministry. You have a Commissioner, you have a Permanent Secretary. So, the hierarchy says, ‘If you want to get something, you have to apply through the Ministry of Health.’ So, the Ministry of Health are in charge of policy. And then they'll tell you, you could go ahead and implement it. So, I believe the right thing to do is to write to the Commissioner through the ministry, then the ministry will tell you, ‘Fine, the thing is applicable.’” (Administrator)*
			iii. At the community by engaging the parents and other community leaders	*“Giving health talks. In church and any other religious [setting]. You can also go through that route because the people tend to listen to them.” (Doctors Focus Group)*
				*“Like the radio. Most of our setting, people that talk to this hospital from the low socioeconomic class. So, the radio is the best according to my opinion.” (Doctors Focus Group)*
				*“Most of them listen to the radio. So, the radio is the best according to my opinion.” (Doctors Focus Group)*
				*“Right from the beginning, as I have said, the Medical Director actually has his management team which all the head of departments are part of. They have management meetings actually every month or twice. So, everybody has to [inaudible] be told exactly this is what is happening and expect so, so, so and so working in this environment and [inaudible]. So, I think right from the start. That's why I said the ministry should be aware, then the MD, then his management, then it drops to staff and then community.” (Administrator)*
	ii. Implement	Inner setting (implementation climate)	Emphasis was on staffing (through employment) and training.	*“If you can employ more people, good. If you say you can employ more nurses, employ more doctors there will be more hands to take and even man somebody permanently.” (Doctors Focus Group)*
			Setting a start date.	
	iii. Adopt	Process (executing)	Willingness of the leadership of the hospital to adopt the program.	*“I believe our management will support this program, even the nursing department will support this program. And since it is coming down to us, we'll also support the program.” (Administrator)*
				*“Because we have already give you the rooms...We have already give all what you want...so we're waiting for you.” (Administrator)*
**MODERATORS**				
	i. External factors	Outer settings		
		□ Patient needs and resources (cost)	The cost of providing care for a child with sickle cell disease was seen as an important aspect of family needs.	*“It affect them morally and economically, because I can say, economically, they will always be in the hospital, spending money for blood, drugs. If they didn't walk to the actual place to look for help. Sometimes they will advise them to go to the medical, traditional medicine, looking for help. Spending money.” (Administrator)*
			Lack of male partner support.	*“Mostly, it's the women, because some of the fathers, they don't even care to come to the hospital with their...their wives.” (Administrator)*
		Cosmopolitanism	External support from NGOs and other support groups like Friends of the Hospital.	*“That why they have associations and some NGO even like one in Badarawa to makes the health care delivery to them, to the cost implication, to lessen it. That's the essence of all these NGO. So that if you go to any private hospital for admission, it's money all the time. But all these NGO whatever, they can form a support group so that....Just like those who are outside, they're HIV...so, they can also do that for the...sickle cell.” (Administrator)*
		□ External policies and incentives	Strengthening the primary health care system.	*“We're only encouraging, because the primary health, we cannot refer the patient from the secondary health facility to the primary health facility....The only thing we normally encourage them is, let's say the antenatal care or the immunization. Then we always encourage them that it's not necessary for them that they should come here. If they have primary health sectors in their places, let them be attending so that at least they will reduce the workload on us. So, by the time they are attending the...if there is anything that is bigger than them, then they will refer them to come to us.” (Administrator)*
	ii. Caregiver	Patients’ needs and resources (education)	How the caregiver understands sickle cell disease, stroke risk, and stroke screening may influence utilization of the program.	*“So, use the language, very simple and clear language that the child or the mother, if it is a very small baby that cannot understand you're saying, you talk to the parents. If it means even bringing both parents, the father and the mother together so that—so that you can be able to at least give them the information so that they will know how to take care of the child.” (Administrator)*
	iii. Teams	Inner setting (Networks and communication; Implementation climate, Tension for change) and Characteristics of individuals.	Some people talked about the importance of all team members having common knowledge about the body and the importance of learning TCD, as well as the consequences of stroke.	*-“Not waiting until we look for somebody that knows how to operate the machine. So, the best thing, like the nurses that are here, everybody should be given the knowledge. If everybody have the knowledge, you will not wait until the person that have the knowledge come and take care of the patient. Once you have the knowledge you will know what to do without even waiting.” (Administrator)*
				*-“Stroke is a very serious medical issue. When you have stroke, you lose function. So, by losing function, it means you're not capable of doing a lot of things, even your thinking, your rationale, your movements, your coordination.” (Administrator)*
	iv. Intervention	Intervention characteristics	The potential barriers to the implementation of the intervention were identified: (1) prior experience, (2) complexity, and (3) cost.	-*“From the start, it will become very difficult. When you're starting something new, but every time there is going to be some adjustment ... So, by the time we are used to it, we'll be able to adjust to combine the two together, but…we are willing to adjust.” (Nurses Focus Group)*
				-*“My question here is the TCD, is it going to be free for the... for the patients? That's very good.” (Nurses Focus Group)*
				-*“So, anything you want to do, you should also consider the financial aspects.” (Administrator)*

### System

For the health care system (inner setting), we identified six interacting themes: (1) clinic characteristics, (2) resource availability, (3) within-setting communication, (4) tension for change, (5) accessibility of information, and (6) implementation climate.

#### Clinic characteristics

Participants discussed how local clinics are organized to provide care and what kind of care is provided. One important feature of clinics is how different disciplines, such as medicine and nursing, are organized and represented. Nurses tended to have rotations, while physicians tended to have primary assignments. One nurse described how task shifting was already occurring in the clinic because of a doctor shortage:

“Now that we have shortage of the medical doctors. Most times, if you come to this hospital on Tuesdays and Thursdays when they see the diabetic and hypertension patients, the nurses are also involved…in seeing the patients, because the doctors that are here cannot cover all the patients…so that nobody will be left behind.” (Nurses’ Focus Group)

#### Resource availability

Many participants discussed a lack of resources. Resource gaps may be due to lack of money, low pay, failure to pay temporary staff, insufficient staffing, and staff turnover. Nurses talked about how high turnover among nurses is created by low pay and how turnover increases caseloads and stress (Table 1).

#### Networks and communications

This refers to the formal and informal communications within an organization. The need to involve stakeholders from the beginning of the program was stressed by one administrator:

“The Medical Director [MD] has his management team, which all the head of departments are part of. They have management meetings every month or twice. So, everybody has to [inaudible] be told exactly this is what is happening and expect … That’s why I said the Ministry should be aware, then the MD, then his management, then it drops to staffs and then community.” (Administrator)

#### Tension for change

Tension for change is the degree to which staff see the current conditions as problematic and in need of change. The greatest tension for change was around staffing. According to one administrator, task shifting was necessary and already being implemented in hospital functioning. Thus, the administrator felt that the hospital would be ready to add the proposed stroke prevention program (Table 1). This nurse was also ready to embrace task shifting:

“Without the task shifting, the workload will be too much on one person, like what we are experiencing here in our facility. We only have one radiologist, but with this task shifting, when others are being trained, you will see that they’ll be able to attend to more number of children, than delaying, waiting for only one radiologist to take care of the investigation or the test.” (Nurses focus group)

#### Accessibility of information

The importance of access to knowledge about SCA and stroke for both the health care providers and parents was highlighted as important in the success of the program and improving the care of children. To be useful, appropriate information about the intervention and the role changes involved in task shifting, along with accurate information for the family’s needs, should be readily available. This nurse stressed the importance of readily available training for nurses on appropriate diagnosis:

“Another challenge is also the accuracy of diagnosing these cases, because of inadequate knowledge or training the nurses acquire (Nurses focus group)”.

#### Implementation climate

This is the willingness of staff to change, the degree to which change is part of the local culture, the recognition of the importance of change, and the extent to which participation in change efforts is encouraged and rewarded. The participants showed evidence of an organizational climate to support change. One nurse discussed the importance of planning for change:

“Like somebody talked about planning, … ….. Even if it’s [your] own housework, if you don’t plan how to do it…at the end of the day, you will leave some things undone. Some simple, simple things that you’re supposed to do, [inaudible] you’ll leave them undone.” (Nurses focus group)

#### Summary of system

The discussion suggested that the local system is burdened by high patient load, lack of funding, low pay, inadequate staffing, staff rotation, and high turnover. Despite these problems, there is evidence that, out of necessity, staff are willing to and are already engaging in task shifting. There is also a commitment to identify changes, including access to training and improved communication, to better meet the needs of patients, providers, and the clinic.

### Individual

The inner circle labeled “individual,” while part of the CFIR, is enhanced by using the theory of planned behavior [24]. Elements of the theory are normative beliefs, control beliefs, and behavioral beliefs. Normative beliefs are the individual provider’s understanding of the behavioral norms (how people are expected to behave) that are part of their institution or profession. Behavioral beliefs are ideas about what to do and how likely different behaviors are to lead to desired outcomes. Control beliefs refer to an individual’s sense of the ease or difficulty of performing different behaviors.

#### Normative beliefs

The discussion of what is usually done focused largely on task shifting the work from doctors to nurses. Doctors, nurses, and administrators agreed the program was important and task shifting was already the norm (see Table 1).

“Definitely, in this hospital normally we practice task shifting. Because in Nigeria, even if you look at the….Not even radiologist, even the ratio of the doctors to the patients—already there’s task shifting in the nurses. And even in radiology, there’s task shifting with radiographers and sonographers, because the amount of patients you see as a radiologist, it’s not possible for you to see.” (Administrator)

#### Behavioral beliefs

Behavioral beliefs were focused on screening procedures and on having an idea of what would be successful. One nurse talked about how to engage caregivers of children with SCA to encourage success (Table 1). Behavioral beliefs also involve understanding what the outcomes or benefits of a choice are. This doctor described the benefits of adopting the screening program:

“Of course, it will reduce the workload of the doctors. That’s the one important benefit I see for the hospital. It will reduce the workload … I think is a plus for the hospital.” (Doctors focus group)

According to administrators, task shifting is the only possible solution. They saw the program as a benefit for the mothers and caregivers of children with SCA, because it provides insight into the disease. One administrator was certain that the stroke prevention program would have positive outcomes for all involved:

“In fact, this is a very good program. It will go a very long way because the patients will benefit from it. The person that is carrying out the work will also benefit from it, because it is a knowledge being given to him. … Also, the hospital will benefit from it because many people would like to come in order to get a better service.” (Administrator)

### Control beliefs

Control beliefs are how confident one is in performing a specific behavior. In the doctors’ focus group, the discussion on control beliefs was on the nurses’ ability to learn and perform the task shifting activities. According to this administrator, the stroke prevention program will work:

“It will work if we put hands together.” (Administrator)

This nurse was confident that nurses would be able to handle task shifting:

“The patient will always be attended to. Because like now, we are always available. The nurses are always on ground” (Nurses focus group)

#### Summary of individual

For the adoption of the stroke prevention program, involved clinicians need to believe that it is the right thing to do, they need to know what to do, and they need to be confident that they can implement the program. There is evidence that at least some of the doctors, administrators, and nurses are ready to adopt the program.

### Implementation process

We conceptualized the implementation process as having three stages; first, an implementation team is *engaged* and works to develop goals, policies, personnel, time schedules, and other details of what the program will look like. This involves planning, leading, and executing [26]. The next step is to *implement* the program. Once a program has been implemented, it may eventually transition to the final stage, which is *adoption*. In the adoption stage, the focus is on sustainability and creating positive outcomes for the prevention strategy.

#### Engage

To implement the proposed stroke prevention program, stakeholders in both the inner and outer setting must be engaged, including doctors, nurses, caregivers, and the community at large. Some of the quotes include:

“The need, especially the pediatricians, I think they should be the frontline.” (Doctors focus group)

“Giving health talks. In church and any other religious gathering. You can also go through that route because the people tend to listen to them.” (Doctors focus group)

There was also discussion of how the Ministry of Health and Commissioner need to be involved:

“This is a policy now; policies are done from the ministry. You have a Commissioner, you have a Permanent Secretary. The hierarchy says, ‘If you want to get something, you have to apply through the Ministry of Health.’ The Ministry of Health are in charge of policy. And then they’ll tell you, you could go ahead and implement it. I believe the right thing to do is to write to the Commissioner through the Ministry, then the Ministry will tell you, ‘Fine, the thing is applicable.’” (Administrator)

#### Implement

The implementation process consists of many parts such as hiring new staff, training existing staff, and reorganizing the way care is provided. One nurse talked about the importance of setting a program start date.

“A date should be fixed for TCD screen. Like the SCD clinic is every Friday. So why not that Friday that they’re coming for the clinic” (Nurses focus group).

#### Adopt

There was limited discussion about adoption of the program, but comments from administrators showed that they were ready to adopt the program.

“I believe our management will support this program; even the nursing department will support this program. And since it is coming down to us, we’ll also support the program.” (Administrator)

#### Summary of implementation process

Participants identified important elements involved in the process of implementing the task shifting stroke prevention program. It is important to engage all stakeholders, including patient families, the government, and other external organizations. Participants identified specific ways to reach the community to ensure that families are aware of the need for stroke prevention and to advertise the program. Creating an implementation team is an important part of planning. Selecting and training the right people is a vital for team development. The implementation process should proceed from engagement to resource development to setting a program start date.

### Moderators

Moderators are factors that influence how the program is planned, implemented, and adopted. Moderators can be either barriers, factors that make the work more difficult or complex, or facilitators, factors that contribute to the success of the work. Four main categories of moderators were identified: external (outer setting), caregiver (outer setting), teams (inner setting), and intervention (intervention characteristics).

#### External

Outer setting factors are things outside of the clinical setting that can have an impact on the planning and implementation of the program. Three main themes were identified: (1) patient needs and resources; (2) cosmopolitanism, referring to external organizations; and (3) policies and mandates. Discussion of the needs and resources of patients largely focused on their financial needs, which is especially salient in Nigeria with a high rate of extreme poverty [27]. One doctor identified having money for transportation as a family need.

“Finance. You can give them money; most of them will come for certain when you give them money for transport” (Doctors focus group)

This administrator talked about different types of people in the community who are considered friends of the hospital and the importance of their involvement in program planning and implementation:

“Friends of hospital, you see the Medical Director (MD), our present MD, they have a committee for friends of hospital. So, I don’t know who are directly now, but [crosstalk] traditional rulers….Yeah, traditional rulers, head of communities, and certain people who work within the hospital as volunteers as friend of hospital” (Administrator)

The relationship between primary and secondary care settings in Nigeria will influence how the program is implemented, according to this administrator:

“We’re only encouraging, because the primary health, we cannot refer the patient from the secondary health facility to the primary health facility….The only thing we normally encourage is, let’s say the antenatal care or the immunization. We always encourage them that it’s not necessary for them [to] come here. If they have primary health centers in their places, let them be attending, so that at least they will reduce the workload on us. So, by the time they are attending the…if there is anything that is bigger than them, then they will refer them to come to us.” (Administrator)

This nurse thought that government policy might be used to support staffing:

“I feel that challenge can be tackled if the government too can step up to improve the manpower shortages in our hospitals.” (Nurses focus group)

#### Caregiver

How the patients’ caregivers understand SCA, stroke risk, and stroke screening may influence utilization of the program. Parents of children with SCA are often stressed and overburdened by their child’s condition. This administrator spoke about the financial and social burden parents face:

“For parents, it is a burden every day, your child is sick, you’re in the hospital and he’s not doing well, and you’re wasting your money, you’re wasting your time, and the child is not improving.” (Administrator)

This nurse spoke about how to support caregivers:

“When a child is being brought with a case of sickle cell, you will see that the parents are restless. The first thing, you reassure the parents. Then the child, if he’s in pain, there are analgesics that you can give, you as a nurse. … So, you give the necessary things; you take care of the child until he is calm.” (Nurses focus group)

#### Teams

Within the inner setting, the composition and training of the team that will plan and implement the prevention program is important. This administrator talked about the importance of all team members having common knowledge about the body:

“There are certain things, you think these are things that you did maybe from basic secondary schools, it’s emphasized, emphasized, emphasized, is not that you don’t do it, but you need to have some background of sciences, of anatomy, of physiology ….How will you know all those things if you don’t comprehend?” (Administrator)

One administrator believed the training should focus on radiology technicians rather than nurses, because there is less turnover among radiology technicians (see Table 1). Another administrator cautioned that not everyone can be trained to task shift:

“They have to know what they are looking for, or what should they look for. If not, it’s like a pilot, everybody you’ll talk to fly a plane, but you have to be regulated. …So, medicine, it’s true I agree with task shifting, but it has its own limitations.” (Administrator)

#### Intervention

Some aspects of the stroke prevention program could create barriers to implementation. Three themes were identified: (1) prior experience, (2) complexity, and (3) cost (see Table 1). Complexity refers to the number of people, departments, and professionals who have to be trained to work together. Cost involves the additional money or resources needed to launch and sustain stroke screening.

#### Summary of moderators

Potential barriers and facilitators that might affect the implementation of task shifting to prevent stroke in children with SCA were identified by participants. External barriers included patient needs and resources along with support, from external organizations, including the Ministry of Health, non-governmental organizations, and friends of the hospital committees. Caregivers of children at risk for stroke due to SCA were seen as stressed, burdened, and in need of well-delivered education about the child’s condition and how to navigate the health care system. Health care teams assigned to task shift must be knowledgeable, well trained, enthusiastic, and hard working. Having some direct experience with SCA can be a facilitator. The intervention will be easier to implement if the hospital and hospital staff have prior experience with task shifting and with screening and triaging high-risk patients. Complexity and cost are potential barriers that may make it difficult to implement and sustain the stroke prevention program.

## Discussion

Our data indicate that task-shifting of TCD screening to nurses in a community hospital is a viable option for expanding the reach for stroke screening among children with SCA, thus addressing the current paucity of personnel with expertise in performing TCD screening, a rate-limiting step in primary stroke prevention in children with SCA, in regions with high burden of the disease [28]. Health care providers and administrators endorsed the importance of stroke prevention among children with SCA and viewed the evidence-based program as a needed opportunity. Generally, health care providers reported that they were ready and willing to adopt task shifting, and administrators stated that the hospital system was overloaded and that task shifting would relieve some burden. Findings from this study are reflected in our conceptual framework derived from the five domains of the CIFR and the theory of planned behavior (Fig. 1). The framework shows the interaction between the health system factors, the individuals that will drive the adoption of the proposed stroke prevention strategy; and the modifying factors that will influence the implementation process.

We selected CIFR because of its multi-level determinants that guide possible implementation strategies that can affect different levels of implementation contexts [13, 14]. CIFR has been successfully used as a determinant framework in understanding stakeholder perspective to task-shifting of hypertension services to nurses in Ghana and Nigeria [15, 16]. The original CIFR largely helped us identify determinants to the implementation of a stroke prevention program for children with SCA in a community hospital in Kaduna, Nigeria. Since our study was conducted, the CIFR was updated with additional constructs to capture potential influence of local attitude and local conditions [29]. Adding these new constructs to study would have expanded on the contextual factors that could affect our implementation process. Nonetheless, we believe that we have identified a rich set of contextual factors that provide a path to move forward in identifying strategies to address the identified determinants in this study.

Both the system and individual themes captured the stress that exists in providing health care in a community hospital where requested medical and nursing services are greater than the capacity to deliver services. High patient volume and staffing shortages strain the system. Meager salaries were identified as one barrier and a common reason for staff turnover. Both doctors and nurses endorsed task shifting TCD screening to nurses. Several providers identified that task shifting was necessary and the only way a limited number of health care providers could adequately care for the large population of individuals with SCD. Successful examples of task shifting in resource constrained settings include nurses managing hypertension in Ghana, who received support from their hospital leadership to effectively control hypertension [15]. Similarly, task shifting in HIV management where the initiation and monitoring of anti-retroviral drugs to people living with HIV was delegated to either nurses or non-clinician physicians provided high-quality, cost-effective care to more patients than a physician-centered model [30].

Within the state of Kaduna and the country of Nigeria, the Ministry of Health has great influence over mandated evidence-based practices. There is a huge deficit in human resources for health in Nigeria. Currently, the World Health Organization estimates four doctors per 10,000 people in Nigeria, compared to 26 doctors per 10,000 people in the USA [31, 32]. These deficits are replicable across other health care personnel, including nurses. Even if Nigeria embarks on emergency training of doctors who will offer stroke prevention to children with SCA, it will take 6 years to certify general medical doctors and another 4 years to train specialists (radiologists, hematologists). Clearly, other alternatives are needed to address this shortage of health personnel. Task shifting, also endorsed by the World Health Organization, provides a viable option to improve health care by making more efficient use of already available human resources [33] while rapidly expanding the human resource pool and building capacity that is more cost effective and sustainable [34, 35]. For this to succeed, all stakeholders should be involved, including the Ministry of Health as a strong mediator of success in the implementation and sustainment process of healthcare initiatives [36, 37].

This study indicated that a limitation in the task shifting strategy from physicians to nurses was inadequate staffing of both professional health care providers. Therefore, task shifting TCD screening to radiology technicians was identified as a viable and potentially more sustainable solution. In high-income countries, TCD screening is routinely performed by certified radiology technologists or sonographers [38, 39]; physicians and nurses rarely perform TCD screening [40]. A facilitator to implementing stroke prevention outlined by our participants was the education provided to health care providers and families. Given the high nursing turnover rates, we anticipate frequent educational sessions and linkage to the established secondary and tertiary health care centers [41]. A systematic approach to training and skills checks must be established to ensure fidelity of TCD screenings. Ideally, a senior staff member would serve as the champion and would also “train the trainer” for future hires. In a systematic review conducted by Joshi et al., training, provision of algorithms, protocols, and guidelines were found to be key enablers in successful task-shifting programs [42–44].

In terms of reaching the families, we are planning in-person advocacy through community meetings in town halls as well as religious gatherings and media campaigns over the radio, on social media, and in other venues to inform caregivers about the importance of TCD screening and stroke prevention. Consistency and bundling of services (i.e., TCD screens conducted during usual SCD clinic days) may facilitate caregiver buy-in. Additionally, engaging members of the community to serve as “champions” is a strategy that can be used in increasing the reach for TCD screening [45]. Several studies have reported that the use of community health workers (also known as lay health workers, outreach workers, patient navigators) in chronic conditions like asthma, hypertension, heart disease, and HIV has improved health outcomes [46, 47]. We anticipate their engagement in creating awareness on SCA and TCD screening for stroke prevention will help improve outcomes [48]. Costs associated with services and the financial burden for families must also be considered for implementation and long-term adaptation of the stroke prevention program; therefore, an extension of TCD screening may be needed in the primary care setting. This would decrease costs for families as they are usually located nearer to them. Serial educational and booster training sessions will be needed to maintain skills of TCD screening in the community hospital or primary care.

## Conclusion

Health care providers and administrators at a community hospital in Kaduna, Nigeria, endorsed the importance of stroke prevention among children with SCA and are open to task shifting for completing TCD, as many providers have already adopted this practice for other needs due to short staffing. Repeated cycles of education will be needed for all levels of the medical team and the families. For these efforts to yield the expected favorable outcomes, the government of Nigeria must recognize the impact of SCA on its population and make deliberate efforts toward improving care for individuals with SCA by enabling policies that will be implemented into routine practice.

### Supplementary Information

**Additional file 1.**

## Data Availability

Available on request.

## Abbreviations

CFIR: Consolidated Framework for Implementation Research
SCA: Sickle cell anemia
SCD: Sickle cell disease
SPRING: Stroke Prevention in Nigeria
TCD: Transcranial Doppler
VU-QRC: Vanderbilt University Qualitative Research Core
