# Supplementary material for: Barriers and facilitators to a task-shifted stroke prevention program for children with sickle cell anemia in a community hospital: a qualitative study
Source: Implement Sci Commun. 2024 Jan 15;5:10. doi: 10.1186/s43058-023-00534-z (PMC10790401; doi:10.1186/s43058-023-00534-z)
Supplement: Supplementary file 1 — Additional file 1. [file 43058_2023_534_MOESM1_ESM.doc]

**FOCUS GROUP DISCUSSION GUIDE – HEALTH CARE PROVIDERS**

**FOCUS GROUP PARTICIPANTS’ PROFILE/BIODATA**

**Group #:**

**Date:**

**Location:**

| **Participant Number** | **Age** | **Sex** | **Marital Status** | **Tribe** | **Professional background** | **Current work & location** | **Years of experience** | **Address** | **Phone number** |
| --- | --- | --- | --- | --- | --- | --- | --- | --- | --- |
|  |  |  |  |  |  |  |  |  |  |
|  |  |  |  |  |  |  |  |  |  |
|  |  |  |  |  |  |  |  |  |  |
|  |  |  |  |  |  |  |  |  |  |
|  |  |  |  |  |  |  |  |  |  |
|  |  |  |  |  |  |  |  |  |  |
|  |  |  |  |  |  |  |  |  |  |
|  |  |  |  |  |  |  |  |  |  |
|  |  |  |  |  |  |  |  |  |  |
|  |  |  |  |  |  |  |  |  |  |
|  |  |  |  |  |  |  |  |  |  |
|  |  |  |  |  |  |  |  |  |  |
|  |  |  |  |  |  |  |  |  |  |
|  |  |  |  |  |  |  |  |  |  |

**Context in beginning**

***(****As the interviewer, introduce yourself, explain the objectives of the interview and obtain the respondent’s individual consent to be interviewed. Note each respondent’s name and job title; describe his or her duties; and enter their current work/institution’s name and location and the date of the interview****.)***

**Interviewer’s welcome, introduction and instructions to participants**

Thank you all for joining us today. Before we begin I just want to review some information with you. As we discussed with each of you as you arrived today, this group will be audio-recorded to ensure we accurately capture the information you share with us here today. Each of you has agreed to be audio-recorded for today’s focus group session. When we report the information we learn from the focus groups, none of your names will be used and these recordings will not be shared with any individuals outside of the research team. XX is here taking notes on what is discussed today to make sure we capture as much of the information shared and the experience in this focus group as possible.

We are now going to turn on the audio recorders and begin our discussion.

<<turn on audio recorders>>

**Introduction:** This focus group discussion is designed to assess your current thoughts and feelings about sickle cell disease, stroke and how these patients are currently managed. We also would like to identify the challenges you think these patients and their families face both at home and in the health facility. We want to know how you think you can help these patients and what you think is important for to be able to help this patient population. Have different experiences that will help in the taking care of these children.

All of the people here have provided care for children with sickle cell disease either in this facility or elsewhere. While there are similarities in your experiences, we will like you all to share as much information as you can with the group. Each of you has a unique experience and point of view, and this is what we want to learn more about today.

There are no right or wrong answers to the questions we ask, we are here to learn about your experiences.

We expect the discussion to take about an hour and a half. We’ll provide you with a break halfway through the session.

**Anonymity:** Despite being recorded, I would like to assure you that the discussion will be anonymous. The records/tapes will be kept safely in a locked facility until they are transcribed word for word, then they will be destroyed. The transcribed notes of the FGD will contain no information that would allow any of you to be linked to specific statements. You should try to answer and comment as accurately and truthfully as possible. I and the other focus group participants would appreciate it if you would refrain from discussing the comments of other group members outside the focus group. If there are any questions or discussions that you do not wish to answer or participate in, you do not have to do so; however please try to answer and be as involved as possible.

**Ground rules**

Other things to remember during this group discussion today is to please be respectful of all the other people in the room and the different experiences shared today. Please also be respectful when others are talking and do not interrupt other people when they are speaking. If possible, please also make sure your cell phones are silenced so they do not interrupt the discussion. If you need to take a call during the discussion, please step outside the room so this doesn’t interrupt the discussion or interfere with the audio recording. When you do have something to say, please indicate by show of hand and your tag number to do so. It is important that we obtain all your views.

***Participant Introductions***

As we get started, we’re going to go around the group to introduce ourselves and I’d like each person to share at least one activity they find meaningful or that is important to you. This can be something you’re able to do right now or it can be something you used to do that is difficult for you now—either or both are fine to share.

I’ll get us started by introducing myself. My name is X and I enjoy Y.

<<Make sure each participant states their name and activities.>>

Thank you all for sharing. As we move forward I want you to keep these activities in mind and we can expand on these topics throughout our discussion today.

Before we start our discussion, please permit me to play a short video clip on our topic for discussion.

1. **Introductory question**

Could you describe your prior experience in providing care to kids with SCD?

- Is anyone happy to share his or her experience with us? (Allow two or three participants to share their experiences as ice breakers)

1. **Characteristics of the individual** (Knowledge and belief about the intervention)
2. Tell me, what do you know about SCD.

- How do you recognise children with SCD?
- How do you think children with SCD should be cared for?

*(Probe; Specialty clinic with prescriptions OR chemists OR homecare?*

- What would be your priorities in providing care and counselling the parents?

*(Probe: Vaccines, Good nutrition, Frequent pain medication? etc*

1. In the video shared in the beginning, we saw stroke screening.

- How would someone be able to diagnose a stroke?

*(Probe: Participant to itemise all possibilities and anything else on how they perceive a child with stroke to look like)*

*If no one answers or no one is correct, just thank them and say you will return with more education*

- How can you help prevent stroke in children with SCD?

*(Probe for reasons why participants think they can (or cannot) prevent stroke in children with SCD) and the ways they believe they can achieve same.*

3. Could you tell me what you know about Transcranial Doppler Ultrasonography (TCD)?

*(Probe: encourage the participants that have heard of TCD to describe TCD and how it is done)*

***<< Explain what TCD is, how it’s done and why and other components of stroke prevention strategy in SCD>>***

***Transcranial doppler ultrasound (TCD) is a procedure performed on children with sickle cell disease to help identify those that are at risk of developing stroke. A small probe (just like a crayon) is used to measure the flow of blood into the brain. Based on the speed of the blood, those that are likely to have a stroke are identified and this stroke can be prevented. Our previous studies have shown that using a medication called hydroxyurea for the children identified to be at risk of stroke will prevent them from having the stroke. So our stroke prevention strategy in children with sickle cell disease include; a) TCD screening; b) identifying those with abnormal TCD values and c) strating these children on hydroxyurea.***

5. Who do you think should perform TCD on children with SCD?

*(Probe: encourage participants to discuss the different categories of persons they think should be performing TCD.*

*6* . How do you feel about non-physicians being trained how to perform TCD?

*(Probe: Do you have any fears about training/ Would you be excited to learn a new skillset?*

**II. Outer settings** (Patients’ needs and resources)

1. What additional information on SCD and stroke do you need to help you provide better care for patients with SCD?

*(Probe: How would it be delivered? Educational sessions?Handouts?Hands on training?Videos?*

1. What patient resources do you think will need to teach patients and care givers.

*(Probe: Encourage the participants to give examples of resources that will help in patient information; e.g. Informational cards on stroke?Videos? Health education sessions?*

1. **Intervention Characteristics**

***<< Give a brief description of task shifting>>***

***Task shifting is defined as the rational redistribution of tasks among health workforce teams, where specific tasks are moved from highly qualified health workers with to HW shorter training and fewer qualifications in order to make more efficient use of available human resources for health. In our case, TCD screening is traditionally the business of the radiologists, but we know we don't have anough rediologists and if we depend on them for TCD, majority of children at risk of developing stroke will not be identified and therefore, they wille end up with a stroke. To address this issue, we want to task shift TCD screening to other health care professionals e.g. nurses or non-specialist doctors.***

- 1. Tell me how you feel about task shifting.

*(Probe: What their personal opinion or preferences are regarding task-shifting of traditional roles to non-physicians/non-health workers?)*

- 1. What benefits do you see with nurses performing TCD for children with SCD in your facility?

(*Probe: Encourage participants to list the benefits and say Why?)*

- 1. What challenges do you see with nurses performing TCD for children with SCD in your facility?

(*Probe: participants to discuss the pros and cons of task shifting of TCD to nurses*)

- 1. What do you think will help to overcome the challenges we have discussed so far?

(*Probe: encourage the participants to proffer solutions to the anticipated problems enumerated earlier) based on the enumerated challenges).*

1. **Inner setting** **(Implementation climate)**

**<<Overtly state that the proposed plan is to use task shifting. Then build concensus to work together…..>>**

***We propose task shifting of TCD screening to nurses. By doing this, we will ensure that more children that are eligible for TCD screening are reached. Further, the TCD will be conducted in the clinics and the doctors and nurses will be able to make quick decisions on the children that may require hydroxyurea. Using this strategy will enable us to work as a team in improving the care of children with sickle cell disease.***

1. How do you think task shifting will fit into your current schedule?

*(Probe: Participants to describe how they will accommodate an extra responsibility)*

1. Tell me how you think you will adapt?

*(Probe: Participant to list and describe each adaptation measure/strategy)*

*(Probe to get details on how they intend to make task shifting more efficient)*

1. **Implementation process** (Planning and engaging)

**<< Explain how the stroke prevention program will be developed, the role of nurses and initiation of hydroxyurea)**

***Implementing the stroke prevention program will involve; i) provision of the required information and materials on stroke to the health care providers and the patients; ii) training nurses to conduct TCD screening; iii) identifying the children at risk of developing stroke; iv) initiation of the free hydroxyurea which has been provided by the government and v) ensuring that this program is sustained through the commitment of all stakeholders.***

1. What would you need to tell/inform parents about the stroke prevention programme?

*(Probe: Details on how parents will be reached should be described)*

1. How could you help the families remember to return for appointments?

*(Probe: Participants to list ways/approaches to getting parents to agree to bring their children for TCD and stroke detection. Give an example of a reminder call or text.).*

3. Tell me how we can inform people in the community of TCD and stroke prevention?

*(Probe: what methods will they use for community advocacy? Will they identify community champions to help with advocacy?)*

**Concluding question**

1. Of all the things we’ve discussed today, what would you say are the most important issues you would like to additionally express/emphasise about SCD, Stroke and Task shifting?
2. Any other recommendation/suggestion on how to task shift and prevent stroke in children with SCD?

**Summarize and validate. Have I missed out anything? Do you have any questions for me/us?**

Thank you for participating. This has been a very useful discussion and we hope you found it interesting. Your opinions will be a very valuable contribution to the study. Please do not hesitate to contact the Principal Investigator or I on any issue related to this subject matter. I would like to remind you that any comments featuring in this report will be anonymous and your privacy will be guarded with utmost confidentiality. Before you leave, please kindly provide me with all information regarding your personal details.

**Thank you for your time and attention!**

**KEY INFORMANT INTERVIEW GUIDE – ADMINISTRATORS**

**KEY INFORMANT BIODATA**

**Please kindly provide the following information (circle or tick the most appropriate options).**

1. Age (in years):…………………………………………………………………………

2. Gender: □ Male □ Female

3. Marital status: □ Single □ Married □ Widowed □ Divorced □ Separated

4. Tribe: □ Hausa □ Yoruba □ Igbo □ Other (specify)…………..

5. Professional background:

□ Nursing

□ Obstetrician

□ Paediatrician/Neonatologist

□ Medical officer

□ Accountant

□ Administrative staff

□ Other: (please specify) ………………………………………………………………………

6. Current position work and location:…………………………………………………………………

7. How many years of experience have you had in this current job?.......................................................

8. Can you briefly describe your roles and responsibilities in this position?;

…………………………………………………………………………………………………………...

…………………………………………………………………………………………………………………………………………………………………………………………………………………………

***Thank you for your response.***

**KEY INFORMANT INTERVIEW GUIDE**

**Facilitator’s welcome, introduction and instruction to key informant**

**Welcome** and thank you for volunteering to take part in this interview. You have been asked to participate as your point of view is important. I realize you are busy and I appreciate your time.

**Introduction:** This interview is designed to assess your current thoughts and feelings about an intervention that will help in improving the care and management of children with sickle cell disease. This intervention involves the introduction of task shifting in the management of sickle cell disease and stroke in children in your facility. The interview will take no more than forty-five minutes. May I tape the discussion to facilitate its re-collection? (if yes, switch on the recorder)

**Anonymity:** Despite being recorded, I would like to assure you that the discussion will be anonymous. The tapes will be kept safely in a locked facility until they are transcribed word for word, then they will be destroyed. The transcribed notes will contain no information that would allow you to be linked to specific statements. You should try to answer and comment as accurately and truthfully as possible. If there are any questions or discussions that you do not wish to answer or participate in, you do not have to do so; however please try to answer and be as frank and involved as possible. You may wish to opt out if you are not interested in participating.

**Ground rules**

- There are no right or wrong answers
- You do not have to speak in any particular order
- When you do have something to say, please do so and stop me to emphasise at any point in time during the interview session.
- Do you have any questions? (Note them and provide answers as much as is possible).

(Once the respondent agrees, proceed with the interview)

**Warm up**

First, I’d like you to introduce yourself and what you do. Can you tell me your name?

**Introductory question**

I am just going to give you a couple of minutes to think about your experience of providing care to children and other persons with SCD. Please kindly share your experience with me.

**Guiding questions (based on the CIFR Framework and Survey Objectives)**

1. **Intervention Characteristics**
2. What do you understand about task shifting?
3. How do you feel about task shifting?
4. Do you think task shifting can be done in your Institution?
5. In what department or units do you think task-shifting will help?
6. Which cadre of staff would you want to adopt task shifting for?
7. Who do you think should be involved in the decision on task shifting?
8. **Characteristics of the individual** (Knowledge and belief about the intervention)
9. Tell me what you know about sickle cell disease?
10. How do you think SCD affects people? (<<what problems do you think they have?>>)
11. Do you know any child with SCD? (>>Describe how he/she looks>>)
12. Did you know that children with SCD could have stroke?
13. Tell me how you think stroke can affect children and their parents/caregivers? >> Intellectually, emotionally, financially?>>
14. How do you think you can help children with SCD and their parents?
15. **Outer settings** (Patients’ needs and resources)
16. Tell me the kind of information you think will help you in addressing the needs of patients with SCD and their caregivers in understanding SCD?
17. Tell me in what ways you think this information can best be delivered or disseminated to patients and their caregivers?
18. Tell me more on how the patients can be engaged in understanding SCD and stroke and how it can be prevented.
19. What problems do you foresee will be encountered in the process of engaging patients regarding SCD and stroke?
20. Do you think other health facilities like other PHCs can be involved? Tell me how?
21. **Inner setting** (Implementation climate)
22. Do you think introducing task shifting will help children with SCD in terms of stroke prevention?
23. Tell me how task shifting aligns with your institution’s vision and mission?
24. How will you implement task shifting? How will you form the team? Where will the personnel be selected from? How soon will the team come on board?
25. Tell me how to introduce task shifting?
26. Tell me the resources you will make available for this process?
27. How will the team communicate with the management?
28. Tell me how you will ensure that task shifting is sustained? << commitment to project based on role>>.
29. **Implementation process** (Planning and engaging)
30. How do you intend to start the stroke prevention programme?
31. Tell me the individuals you think are key stakeholders at this time. <<Are you going to assign? Or allow them to volunteer? >> What characteristics will you be looking out for?
32. Tell me how you will ensure staff support the programme.
33. Tell me how you will ensure the programme is continued.
34. How will you get the buy-in of staff within your organisation?
35. Tell me how you will identify champions within the organisation.
36. Tell me how you intend to engage them.

**Concluding question**

- Of all the things we discussed today, what would you say are the most important issues you would like to express about SCD, stroke and task shifting?

**Conclusion**

Thank you for participating. This has been a very useful discussion and we hope you found it interesting. Your opinions will be a very valuable contribution to the study. Please do not hesitate to contact the Principal Investigator or I on any issue related to this subject matter. I would like to remind you that any comments featuring in this report will be anonymous and your privacy will be guarded with utmost confidentiality. Before you leave, please kindly provide me with all information regarding your personal details.

**Thank you for your time and attention!**
